# Bidirectional Role of β2-Adrenergic Receptor in Autoimmune Diseases

**DOI:** 10.3389/fphar.2018.01313

**Published:** 2018-11-27

**Authors:** Li Wu, Yu Tai, Shanshan Hu, Mei Zhang, Rui Wang, Weijie Zhou, Juan Tao, Yongsheng Han, Qingtong Wang, Wei Wei

**Affiliations:** ^1^Key Laboratory of Anti-Inflammatory and Immune Medicine, Ministry of Education, Collaborative Innovation Center of Anti-Inflammatory and Immune Medicine, Institute of Clinical Pharmacology, Anhui Medical University, Hefei, China; ^2^Department of Emergency Medicine, The First Affiliated Hospital, University of Science and Technology of China, Hefei, China

**Keywords:** autoimmune diseases, β2-adrenergic receptor, single nucleotide polymorphisms, immune response, soft regulation

## Abstract

Disorder of the sympathetic nervous system (SNS) is closely related to the pathogenesis of various autoimmune diseases (ADs). Catecholamine triggered beta2-adrenergic receptor (β2-AR) signaling is important in creating a bidirectional response in the progression of ADs due to factors including diverse expression patterns, single nucleotide polymorphisms (SNPs), biased signals, and desensitization of β2-AR, as well as different subtypes of Gα binding to β2-AR. In this review, we summarize the actions of β2-AR signaling in regulating the functions of immunocytes and in the pathogenesis of ADs, and the application of β2-AR agonists or antagonists in treating major types of ADs is also discussed. We suggest that restoring the immune balance via a soft regulation of the expression or activation of β2-AR is one of the promising therapeutic strategies for systematic ADs.

## Introduction

Although the current understanding of the pathogenesis of autoimmune diseases (ADs) such as systemic lupus erythematosus (SLE), rheumatoid arthritis (RA), myasthenia gravis (MG), and Grave’s disease (GD) is unsettled; many ADs hold in common the expression of autoantigens, abnormal immunoregulation, and shared genetic factors (Harris et al., 2018). Researchers have found that the nervous system is an important regulator in the function of immune cells and thus affects inflammation important in the pathogenesis of ADs (Weissert, 2017; Bellinger and Lorton, 2018). Elevated pro-inflammatory cytokines signal to brain and influence the activity and reactivity level of sympathetic nerves (Kenney and Ganta, 2014). Chronic inflammation in RA is accompanied by activation of the sympathetic nerves system (SNS) and relative parasympathetic hypofunction (Lowin and Straub, 2015). This imbalanced autonomic nervous system is a consistent feature of RA patients. Both central and peripheral immune organs innervated precisely by sympathetic nervous (Bellinger and Lorton, 2018). Activated sympathetic nervous secrete abundant epinephrine (E) and norepinephrine (NE), which activate α-adrenergic receptors (α-ARs) and β-ARs in immune cells to regulate immune response (Janig and Green, 2014). Literatures reported that β-ARs, which include β1-AR, β2-AR, and β3-AR play important roles in inflammation. Of note, β2-AR is regarded to play a key role in the process of immunological imbalance (Chavan and Tracey, 2017). β-ARs belong to the seven transmembrane G-protein coupled receptors (GPCRs). β1-AR couples with Gαs, while β2-AR is able to couple both Gαs and Gαi. The transition in G-protein coupling from Gαs to Gαi, thus influences cAMP production within the β2-AR microenvironment (Fu et al., 2014). This review will summarize the effects of β2-AR in regulating the function of immunocytes and in the pathogenesis of Ads, with special emphasis on how β2-AR can exert bidirectional function depending on the stage of the disease.

## Regulatory Effects of β2-AR on Immune Cells

β2-AR is widely expressed, with distinct densities, on various immune cells, including T cells, B cells, dendritic cells (DCs), and macrophages (Kolmus et al., 2015). Evidence amply confirms its role in immunomodulation. However, during the different processes of immune diseases, β2-AR exerts contradictory effects on immunocytes (Pongratz and Straub, 2013).

### T Cells

β2-AR expression is predominant in human and murine T cells when comparing with other immune cell subtypes (Ross et al., 2018). Studies suggest that CD8^+^ cytotoxic T cells (Tc) express a significantly higher level of β2-AR compared to CD4^+^ helper T cells (Th). Therefore, stimulation of β2-AR reduced the percentage of interferon-γ (IFN-γ) ^+^ Tc to a much higher extent than that of IFN-γ ^+^ Th (Zalli et al., 2015). Moreover, the level of β2-AR on memory Tc is further increased than that on naïve Tc, leading to a more sensitive response of memory Tc to catecholamine stimulation manifesting as a decrease in cytokine production (Slota et al., 2015). Thus, treatments that activate β2-AR achieve their immunoregulatory effects primarily through inhibiting the cytokine secretion ability and the cell killing function of Tc, as well as some natural killer (NK) cells which also express CD8. Stimulation of β2-AR reduces the production of interleukin-2 (IL-2), granulocyte-macrophage colony-stimulating factor (GM-CSF), IFN-γ, and IL-3; yet, it does not influence IL-4 level (Grailer et al., 2014) in murine T cells. However, in human T cells, β2-AR signals to IL-2 production facilitates the Th2 cell differentiation and recues the Th1/Th2 balance under the circumstances of inflammation which indicates that the β2-AR function is species dependent (Scanzano and Cosentino, 2015). Even from the same species, the T cells from different tissues are diversely regulated by β2-AR. Chronic β2-AR stimulation differentiates cord blood T cells to Th2 cells, while it cannot promote the differentiation of peripheral blood T cells (Padro and Sanders, 2014). β2-AR agonist terbutaline exacerbates anti-CD3/anti-CD28-induced IL-17A production but reduces IFN-γ-secreting Th1 cells, suggesting that β2-AR plays a reciprocal role in modulating human Th1/Th17 balance (Carvajal Gonczi et al., 2017).

Interestingly, β2-AR attenuates inflammation and immunization by restricting T cells in immune organs (Tracey, 2014). Activation of IL-13^+^ human memory T cells by β-AR agonist immediately increases the production of cyclic adenosine monophosphate (cAMP) and the activity of protein kinase A (PKA), leading to reduced p38 mitogen-activated protein kinase (MAPK) activation. Subsequently, p38 MAPK inhibits CD3-induced CD25 expression and CD3-mediated IL-13, IFN-γ, and IL-2 production (Lajevic et al., 2011). Calcimycin and phorbol myristate acetate (PMA)-triggered p38 MAPK, ERK, and nuclear factor-κB (NF-κB) activation is biasedly inhibited by β-agonist leading to selective reduction of IFN-γ and IL-2 production but not IL-13 (Loza et al., 2006). Therefore, the impaired function of β2-AR in circulating T cells may induce immunological diseases such as RA by decreasing production of select cytokines (Kenney and Ganta, 2014).

### B Cells

Autoantibodies produced by B cells are widely observed in the majority of ADs. When activated, β2-AR initially couples with Gαs to promote physiological production of cAMP and inhibit the proliferation of human peripheral B cells (Faisy et al., 2010). However, the expression of β2-AR is reduced on B cells in RA patients, leading to the abnormal survival of activated B cells and the accelerating progress of disease. Catecholamines including E and NE are reported to have a positive effect on attenuating specific mitogens that mediate B cell proliferation. This function can be abolished by β2-AR antagonists (Hu et al., 2013). Therefore, exciting the autonomic nervous system is regarded as a promising therapeutic strategy for treating RA (Ulloa et al., 2017).β2-AR signaling impairs IL-17 receptor induced maturation and anti-collagen II autoantibody production of B cells in mice with collagen induced arthritis (CIA) (Pongratz et al., 2014). Researchers also reported that activation of β2-AR by NE accelerates antibody responses of B cells upon immune challenge (Simkins et al., 2014). Antigen exposed B cells or β2-AR agonist terbutaline treated B cells express higher level of CD86 than resting B cells, combining with CD86 stimulation, IgG1 and IgE production is obvious increased in IL-4 dependent manner. β2-AR induces CD86 expression through cAMP-PKA pathway (Qiao et al., 2018). Later on, people found that combined stimulation of CD86 and β2-AR increases IgG1 secretion of human B cells through the promotion of transcription of Oct-2 and its coactivator OCA-B (Podojil and Sanders, 2005). When ADAM10 gene transcription and CD23 expression is enhanced by the activation of β2-AR, the upregulated CD23 and ADAM10 can be shuttled to exosomes and stimulate IgE production of recipient primed B cells (Padro et al., 2013). In addition, β2-AR signaling promotes the expression of IgE and its regulator soluble CD23 through downstream PKA and p38 MAPK pathways in B cells (Sergienko et al., 2012). Taken together, these studies portray a picture where a reduction in β2-AR signaling in B cells of individuals with AD is associated with unchecked B cell proliferation, increased autoantibody production, and a reduction in immune response upon challenge. Moreover, the different mechanisms on B cell activation and isotype switching provide a clinical therapeutic target in IgG1 or IgE response-mediated diseases.

### Dendritic Cells

Dendritic cells (DCs) express α1-AR, α2-AR, β1-AR and β2-AR. Nevertheless, β2-AR is the primary effecter of DCs for cytokine production and antigen presentation, with β2-AR able to exert either a stimulatory or inhibitory function that is dependent on the differentiation status of DCs and/or the activation time of the receptor (Wu et al., 2016). Activation of β2-AR with the selective agonist clenbuterol restrains human DCs differentiation from monocyte (Giordani et al., 2015). In differentiated DCs, *in vitro* treatment with isoproterenol (ISO) reduced CD86 and MHC-II expression, enhanced antigen uptake and IL-10 production, and simultaneously prevented T cells activation and pro-inflammatory cytokines secretion such as TNF-α in a β2-AR dependent manner (Wu et al., 2016). In another study, stimulation of β2-AR by NE induced the migration of DCs from antigen sites to immune organs but suppressed the antigen presentation capacity of DCs to activate T cells. In detail, the stimulation of β2-AR on CD11c^+^CD8a^+^ DCs impaired DC mediated CD8^+^ naïve T cell activation (Nijhuis et al., 2014). Meanwhile, β2-AR signaling in DCs reduces IL-12 production via inhibition of the activation of NF-κB pathway. Decreased IL-12 leads to a shift in the balance of IL-12/IL-13 in LPS induced DCs, preventing IFN-γ production and Th1 development while, increasing IL-17 levels, thus affecting adaptive immunity (Takenaka et al., 2016). Stimulation of β2-AR also decreases the level of IL-1, IL-6, and TNF-α through attenuating toll-like receptor (TLR) response (Manni et al., 2011). Therefore, the major effect of β2-AR stimulation in DCs is a reduction in the number of T cells activated which correlates with a reduction in cytokine release from DCs.

### Macrophages

Accumulating evidence shows that β2-AR signaling plays a pivotal role in macrophage activation and pro-inflammatory cytokine production. Some researchers reported that stimulation of β2-AR inhibits the activation of RAW264.7 (a mouse macrophage cell line) and THP-1 (a human monocytic cell line) induced by LPS through restraining ERK1/2, JNK and NF-κB pathways (Sharma et al., 2017). Further studies have revealed that β2-AR attenuates ERK activation in a cAMP-dependent manner, and blunts NF-κB pathways via increasing β-arrestin2 expression which is a scaffold protein for IκBα activation (Keranen et al., 2017). The up-regulated cAMP upon β2-AR response triggers cAMP-response element binding protein (CREB), CCAAT/enhancer binding protein beta (C/EBPβ), and activating transcription factor (ATF) signaling. This results in the polarization of macrophages to M2, which is an anti-inflammatory subset (Lamkin et al., 2016). Cyclic-AMP induced expression of mitogen-activated protein kinase phosphatase 1 (MKP-1), an anti-inflammatory factor, also contributes to the negative regulation of macrophages (Levine et al., 1988). Therefore, exciting β2-AR induces the differentiation of microphages into M2 subtype and promotes expression of type 2 cytokines, including IL-6 and TGF-β while simultaneously decreasing the production of pro-inflammatory cytokines such as TNF-α (Grailer et al., 2014). Similarly, β2-AR signaling decreases IL-6, IL-1β and TNF-α in human macrophages derived from monocyte (Ağaç et al., 2018). Nonetheless, β2-AR signaling evokes the secretion of the tumor necrosis factor (ligand) superfamily, member 11 (RANKL) therefore initiating the differentiation of osteoclasts (Jiao et al., 2015).

### Other Immune Cells

β2-AR exerts complicated effects on immune cells and influences the function of almost all kinds of immune cells. Stimulation of β2-AR on peripheral blood mononuclear cells (PBMCs) inhibits interferon alpha-1 (IFNA1) production mediated by Toll like receptor 9 (TLR9) (Hilbert et al., 2013). It drives leukocyte migration and tissue infiltration via enhancement of chemokine production (Grisanti et al., 2016). β2-AR signaling is involved in the reduction of NK cells and inhibits their cytotoxicity (De Lorenzo et al., 2015). But conflicting evidence shows that the sympathetic response attracts mature and activated NKs to peripheral blood and primes innate immunity (Bigler et al., 2015). Taken together, the apparent conflicting functions of β2-AR within the immune system lead to the controversial role of β2-AR in the onset and development of ADs.

## The Role of β2-AR in the Pathogenesis of ADs

The SNS is activated in ADs and acts on humoral and cellular immunity primarily through β2-AR. Having a better understanding of β2-AR in the course and progression of ADs will help to develop β2-AR targeted drugs for the treatments of ADs (Zhao et al., 2011).

### Rheumatoid Arthritis

RA is a long-lasting autoimmune disorder that primarily affects joints. Evidence reveals that the polymorphisms of β2-AR determine the susceptibility of RA. The studies report single nucleotide polymorphisms (SNPs) at codon 16 together with the HLA-DRB1^∗^04 mutation have a positive correlation with the expression of anti-cyclic citrullinated peptide (CCP) and are strongly associated with RA (Malysheva et al., 2008). Similarly, in northern Sweden, RA susceptibility and activity is higher in patients with variants at Arg16 or Gln27 (Xu et al., 2005). Generally, in RA patients, the expression of β2-AR is decreased on both peripheral blood lymphocytes (PBLs) and synovial fluid lymphocytes (SFLs), especially on SFLs (Wahle et al., 2002). Therefore, β2-AR response is significantly attenuated in patients with RA. Therefore, β2-AR induced B cell death is impaired, which then leads to the elevated production of autoantibodies and the progress of RA (Sanders, 2012). As a further result, T cells fail to shift to the Th2 subtype in response to catecholamines stimulation, causing decreased levels of anti-inflammatory cytokines (Lubahn et al., 2014).

**FIGURE 1:**
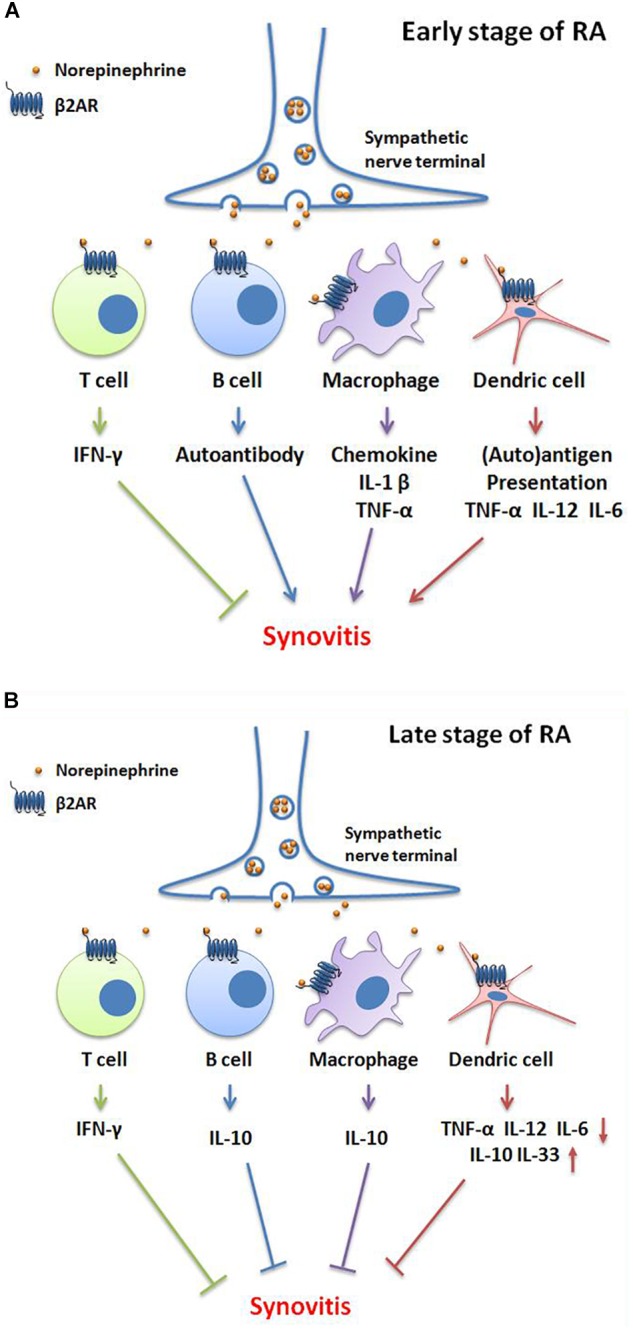
Bidirectional functions of β2-AR signaling in different stages of RA. **(A)** In the early stage of RA, norepinephrine-mediated-β2-AR signaling promotes IFN-γ production of T cells, which is an inhibitory cytokine in RA. However, β2-AR facilitates autoantibody secretion by B cells; chemokine, IL-1β and TNF-α production by macrophages; and auto-antigen presentation, TNF-α, IL-12 and IL-6 generation by dendric cells (DCs), consequently aggravates synovitis and clinical symptoms. **(B)** In the late stage of RA, the activation of β2-AR induces IFN-γ production by T cells; IL-10 generation by B cells and macrophages; decreases the secretion of TNF-α, IL-12, and IL-6 and, increases the IL-10 and IL-33 levels by DCs. These changes then attenuate synovitis and clinical symptoms.

Results indicate that β2-AR desensitization of the β2-AR-AC-cAMP transmembrane signal transduction pathway plays a crucial role in the ongoing inflammation of RA (Zhao et al., 2011). However, β2-AR signaling is observed to exert different functions during the onset of pathogenesis of RA. In the early stages of RA, catecholamines are released from the sympathetic nerve terminal and stimulate β2-AR on T cells, promoting production of IFN-γ, which is a beneficial target for treating RA (Lee et al., 2017). Nevertheless, when catecholamines bind to β2-ARs on other immune cells, they exacerbate the inflammation. For example, β2-AR stimulation promotes B cell autoantibody secretion; drives macrophages to generate various chemokines and pro-inflammatory cytokines including IL-1β and TNF-α; and enhance DC capacity for autoantigen uptake and processing, as well as TNF-α, IL-12, and IL-6 production. At first glance it would appear that β2-AR signaling produced from conflicting roles in regulation of immunoreactions in different cells might cancel each other out, but actually the result is that overall β2-AR signaling accelerates the development of inflammation in early stages of RA. In late stage RA, β2-AR primarily exerts immunosuppressive action by facilitating IFN-γ production from T cells; IL-10 secretion from B cells and macrophages; and IL-10, IL-33 expression from DCs. Moreover, it restrains DC generated inflammatory cytokines such as TNF-α, IL-12, and IL-6 (Pongratz and Straub, 2013) (Figure 1). These studies indicate that the divergent role of β2-AR is cell type and time point dependent.

### Systemic Lupus Erythematosus

SLE is a very different disease from RA. Clinical observations indicate that psychological stress, which often stimulates the SNS, aggravates the activity of SLE (Morand, 2018). The up-regulation of β2-AR on PBMC from SLE patients in response to stress is significantly abolished when compared to healthy subjects (Pawlak et al., 1999). As a consequence, the increment of cAMP in B cells upon β2-AR activation is significantly reduced in SLE patients (Wahle et al., 2001). In addition, β2-AR stimulation exacerbates the production of serum anti-DNA antibodies in MRL/1 pr mice (Chedraoui et al., 2008). Therefore, blockage of β2-AR signaling on B cells is able to successfully reduce the level of immunoglobulin, and attenuates SLE (Hudson et al., 2005).

### Multiple Sclerosis

Multiple sclerosis (MS) is a potentially disabling disease of the central nervous system (Giovannoni, 2017). The mechanisms of progressive focal inflammatory demyelinating lesions is complex, but basically due to the functional loss of β2-AR, which is important in facilitating glycogenolysis and reducing inducible nitric oxide synthase (NOS2) in astrocytes (Cambron et al., 2012). The impaired β2-AR signaling leads to metabolic disorder in axons, the unbalanced distribution of potassium in astrocytes, and an increase in calcium concentration in astrocytes. The transformation of astrocytes to antigen-presenting cells that ensues triggers inflammation, as well as glutamate excitotoxicity, which results in the progression of damage to the myelin sheath (Durfinova et al., 2014). The expression of β2-AR is found to be upregulated on PBMCs of MS patients, but does not change the immune response of T cells (Giorelli et al., 2004). Stimulation of β2-AR on astrocytes promotes intracellular cAMP production and PKA activation, inhibits the transcription of coactivator class II transactivator (CIITA) and IFN-γ-induced major histocompatibility (MHC) class II molecule, thus suppressing the antigen presenting activity of astrocytes and finally reducing the focal inflammatory demyelinating lesions (De Keyser et al., 2010). Thus, measures that enhancing the expression of β2-AR by IFN-β administration or exercise, or exciting β2-AR response by agonists may be considered as promising strategies in treating MS. A total population-based case-control study performed by Tsai et al. (2014) revealed that the β2-AR agonist, fenoterol significantly improved outcomes associated with MS, and reduced the risk of MS by 51%.

### Myasthenia Gravis

MG has the characteristic of formation of autoantibodies to acetylcholine nicotinic receptors at the neuromuscular junction of skeletal muscles (Nacu et al., 2015). The expression of β2-AR on PBMC of MG patients is reduced leading to the attenuated signaling transduction. Thus, stimulation of β2-AR effectively ameliorates many symptoms of MG induced by anti-muscle specific kinase (Ghazanfari et al., 2014). In addition, autoantibodies can also target the second extracellular loop of β2-AR (residues 172–197), which amplifies the β2-AR reactive T and B cells and involves in the patho-genesis of MG. The risk of production of β2-AR autoantibodies in MG patients is increased if SNPs of Arg16Gly on β2-AR are present (Lantsova et al., 2013). Evidence also shows similar polymorphisms of β2-AR affect the incidence of many kinds of ADs such as RA, juvenile idiopathic arthritis (JIA), and GD, the same as MG. The homozygosity for Gly16 can be applied to predict the tolerance of β2-AR agonist treatment, again suggesting that this SNP plays a key role in AD pathology (Lantsova et al., 2013; Wang et al., 2017).

### Grave’s Disease

GD is an AD that affects the thyroid. Although the pathogenesis of GD is still unclear, genetic predisposition, especially for SNPs within β2-AR is regarded to be important in the development of GD (Park et al., 2017). Multiple studies have revealed that the SNPs 47A– > G (Arg16Gly) and 79C– > G (Gln27Glu) on chromosomal region 5q31-33 in β2-AR as well as rs1042714 SNPs contribute to the susceptibility of GD in a Caucasian population. However, these polymorphisms have no significant correlation to the risk of GD in Chinese and Korean male populations (Jazdzewski et al., 2007). Differences in how specific SNPs in β2-AR affect the outcomes of GD in different racial groups highlight the probability that GD, as well as other ADs, are polygenic disorders which need more study to identify important genetic factors that can be targeted for treatment.

### Other ADs

Genomic variations contribute to the morbidity of various ADs besides those discussed above. The significant prevalence of homozygous Gly16Arg genotype in β2-AR has been found to also have a positive correlation with the predisposition to interstitial cystitis (Sugaya et al., 2002). Conversely, the variation of β2-AR does not influence susceptibility to JIA, even though it has similar symptoms to RA (Pont-Kingdon et al., 2009). In JIA, catecholamine response is still abnormally changed. For instance, β2-AR stimulation reduces cAMP level, probably due to its faster degradation (Kavelaars et al., 1998). The divergent effects of β2-AR perturbation in these different ADs underscore the bidirectional nature of this important receptor.

## Targeting β2-AR Treatments of Ads

Catecholamine has both pro- and anti-inflammatory effects in patients with chronic inflammatory disease (Woo et al., 2014). Briefly, by exciting β2-AR, it attenuates type 1 ADs (characterized for cellular immunity) and aggravates the symptoms of type 2 ADs (characterized for humoral immunity) by exciting β2-AR (Namazi, 2004). So far, β2-AR-specific agonists (including salbutamol, metaproterenol, terbutaline, salmetero, ect) or antagonists (such as propranolol, butoxamine, ICI 118551, and so on) have been investigated in treating ADs with inconsistent results probably due to the non-synonymous SNPs of β2-AR, desensitization, or the Gαi/Gαs coupling switch of this receptor in the disease stage (Tandale et al., 2016). The infusion of adrenaline was shown to be a safe and valid method to induce immunological changes in RA patients, with patients presenting an increase in both IL-8- and IL-10-producing monocytes after infusion (Namazi, 2004). In animal models, systemic treatments with a β2-AR agonists like salbutamol or terbutaline is only effective in reducing disease severity when administered starting at disease onset of CIA (Pongratz and Straub, 2014). Correspondingly, the β2-AR agonist fenoterol improves outcomes associated with MS (Tsai et al., 2014).

Conversely, during the initiation phase of adjuvant arthritis (AA), selective β2-AR antagonist butoxamine or ICI 118, 551 induces CD4^+^ Th0 cells to develop into Th1 cells by blocking the inhibitory effects of endogenous NE on Th1 cell development. β2-AR blockade effectively shifts the immune response to the arthritogenic antigen toward a cell-mediated response, that is beneficial for the processing of the arthritogenic antigen. Consequently, blocking β2-AR function during AA development can reduce pro-inflammatory cytokines production via inhibition of the expression and activation of α1-AR, which is elevated by chronic β2-AR stimulation. Therefore, β-blockers decrease arthritic severity in the active phase of AA (Koopman et al., 2011).

Likewise, the application of salbutamol is able to attenuate the rat model of experimental allergic encephalomyelitis (EAE) by decreasing the average neurologic function score of model rats, reducing calpain activity in brain tissues, and rescue the activities of the BB isoenzymes of creatine kinase and catalase (Zhang et al., 2015). Similarly, the blockage of β2-AR using carvedilol protects rats with experimental autoimmune myocarditis (EAM) by ameliorating systolic and diastolic heart function, decreasing the serum level of IL-1β and TNF-α, and increasing IL-10 expression (Liu et al., 2010). More research investigating the effects of β2-agonists or β2-blockers on ADs are listed in Table 1.

**Table 1:** Effect of β2-agonist or β2-blocker on ADs.

Category	Name of the drug	Disease model	Outcome	Reference
Agonist	Terbutaline	Adjuvant challenge of AA	Exacerbate disease pathology	Lubahn et al., 2014
Agonist	Terbutaline	Established AA	Attenuate inflammation	Lubahn et al., 2014
Agonist	Salbutamol	AA	Great clinical benefit by inducing oral tolerance	Cobelens et al., 2002
Agonist	Salbutamol or Terbutaline	Onset of CIA	Reduce disease severity	Cobelens et al., 2002
Agonist	Salbutamol or Terbutaline	Established CIA	Suppress the clinical progression of arthritis	Pongratz et al., 2014
Agonist	Salbutamol	Antibody-induced arthritis	Prevent joint destruction via selective mast cell silencing	Kneilling et al., 2007
Agonist	Salbutamol or Terbutaline	EAE	Decrease the average neurologic function score	Zhang et al., 2015
Agonist	Fenoterol	MS	Reduce the risk	Tsai et al., 2014
Agonist	Albuterol	Patients with MS	Well tolerated and improves clinical outcomes	Khoury et al., 2010
Agonist	Salbutamol	Secondary progressive MS patients	Be in the treatment of MS	Makhlouf et al., 2001
Agonist	Terbutaline	MG patients	An effective adjunct therapy	Soliven et al., 2009
Agonist	Albuterol	Anti-MuSK MG	Reduce whole-body weakness	Ghazanfari et al., 2014
Agonist	Formoterol or Salbutamol	EAM	Suppress the development of EAM	Nishii et al., 2006
Agonist	Salbutamol or Albuterol	SLE	Improve the shrinking lung syndrome	Munoz-Rodriguez et al., 1997
Antagonist	Propranolol	Hypertensive patients with type 2 ADs	Beneficial	Namazi, 2004
Antagonist	Butoxamine or ICI 118, 551	Initiation phase of AA	Deteriorate the symptoms	Lubahn et al., 2014
Antagonist	Butoxamine or ICI 118,551	Established AA	Significantly retarded disease onset and reduced the severity of joint injury	Levine et al., 1988

*AA, adjuvant arthritis; CIA, collagen induced arthritis; EAE, experimental allergic encephalomyelitis; MS, multiple sclerosis; MG, myasthenia gravis; EAM, experimental autoimmune myocarditis; ADs, autoimmune diseases.*

## Conclusion

Taken together, β2-AR signaling is involved in the pathogenesis and progress of ADs, and is a potential drug target in treating these diseases. However, the genetic polymorphisms and the expression of the receptor is diverse from individual patients or different disease stages. Meanwhile, the function of β2-AR signaling is bidirectional between different species, different immune cell isotype, and different time point of diseases. In a particular situation, β2-AR aggravates inflammation, but in some cases, it reduces the production of pro-inflammatory cytokines and auto-antibodies, acts as an immunosuppressor. Therefore, there is a need for thorough elucidation of the precise regulation of β2-AR in each specific AD (Kohr et al., 2011). In addition, soft regulation of the expression or activation of β2-AR that restores the immune balance will definitely be beneficial to the systematic immune diseases (Wei, 2016).

## Author Contributions

LW wrote the text. YT, MZ, and RW collected references. WZ and JT drew the cartoon. YH wrote the table. QW and WW revised the article. QW designed the topic.

## Conflict of Interest Statement

The authors declare that the research was conducted in the absence of any commercial or financial relationships that could be construed as a potential conflict of interest.

## References

[B1] Ağaç D. Gill M. A. Farrar J. D. (2018). Adrenergic signaling at the interface of allergic asthma and viral infections. *Front. Immunol.* 9:736. 10.3389/fimmu.2018.00736 29696025 PMC5904268

[B2] Bellinger D. L. Lorton D. (2018). Sympathetic nerve hyperactivity in the spleen: causal for nonpathogenic-driven chronic immune-mediated inflammatory diseases (IMIDs)? *Int. J. Mol. Sci.* 19:E1188. 10.3390/ijms19041188 29652832 PMC5979464

[B3] Bigler M. B. Egli S. B. Hysek C. M. Hoenger G. Schmied L. Baldin F. S. (2015). Stress-induced in vivo recruitment of human cytotoxic natural killer cells favors subsets with distinct receptor profiles and associates with increased epinephrine levels. *PLoS One* 10:e0145635. 10.1371/journal.pone.0145635 26700184 PMC4689586

[B4] Cambron M. D’haeseleer M. Laureys G. Clinckers R. Debruyne J. De Keyser J. (2012). White-matter astrocytes, axonal energy metabolism, and axonal degeneration in multiple sclerosis. *J. Cereb. Blood Flow Metab.* 32 413–424. 10.1038/jcbfm.2011.193 22214904 PMC3293127

[B5] Carvajal Gonczi C. M. Tabatabaei Shafiei M. East A. Martire E. Maurice-Ventouris M. H. I. Darlington P. J. (2017). Reciprocal modulation of helper Th1 and Th17 cells by the β2-adrenergic receptor agonist drug terbutaline. *FEBS J* 284 3018–3028. 10.1111/febs.14166 28710773

[B6] Chavan S. S. Tracey K. J. (2017). Essential Neuroscience in Immunology. *J. Immunol.* 198 3389–3397. 10.4049/jimmunol.1601613 28416717 PMC5426063

[B7] Chedraoui A. Uthman I. Abbas O. Ghosn S. (2008). Adrenergic urticaria in a patient with anti-double-stranded DNA antibodies. *Acta Derm. Venereol.* 88 263–266. 10.2340/00015555-0435 18480926

[B8] Cobelens P. M. Kavelaars A. Vroon A. Ringeling M. van der Zee R. van Eden W. (2002). The β_2_-adrenergic agonist salbutamol potentiates oral induction of tolerance, suppressing adjuvant arthritis and antigen-specific immunity. *J. Immunol.* 169 5028–5035. 10.4049/jimmunol.169.9.5028 12391218

[B9] De Keyser J. Laureys G. Demol F. Wilczak N. Mostert J. Clinckers R. (2010). Astrocytes as potential targets to suppress inflammatory demyelinating lesions in multiple sclerosis. *Neurochem. Int.* 57 446–450. 10.1016/j.neuint.2010.02.012 20178822

[B10] De Lorenzo B. H. De Oliveira Marchioro L. Greco C. R. Suchecki D. (2015). Sleep-deprivation reduces NK cell number and function mediated by beta-adrenergic signalling. *Psychoneuroendocrinology* 57 134–143. 10.1016/j.psyneuen.2015.04.006 25929826

[B11] Durfinova M. Bartova R. Prochazkova L. Balco M. Liska B. Gavurnikova G. (2014). Role of astrocytes in pathogenesis of multiple sclerosis and their participation in regulation of cerebral circulation. *Neuro Endocrinol. Lett.* 35 666–672. 25702293

[B12] Faisy C. Pinto F. M. Blouquit-Laye S. Danel C. Naline E. Buenestado A. (2010). beta2-Agonist modulates epithelial gene expression involved in the T- and B-cell chemotaxis and induces airway sensitization in human isolated bronchi. *Pharmacol. Res.* 61 121–128. 10.1016/j.phrs.2009.08.003 19683054

[B13] Fu Q. Xu B. Liu Y. Parikh D. Li J. Li Y. (2014). Insulin inhibits cardiac contractility by inducing a Gi-biased beta2-adrenergic signaling in hearts. *Diabetes Metab. Res. Rev.* 63 2676–2689. 10.2337/db13-1763 24677713 PMC4113065

[B14] Ghazanfari N. Morsch M. Tse N. Reddel S. W. Phillips W. D. (2014). Effects of the ss2-adrenoceptor agonist, albuterol, in a mouse model of anti-MuSK myasthenia gravis. *PLoS One* 9:e87840. 10.1371/journal.pone.0087840 24505322 PMC3914858

[B15] Giordani L. Cuzziol N. Del Pinto T. Sanchez M. Maccari S. Massimi A. (2015). beta2-Agonist clenbuterol hinders human monocyte differentiation into dendritic cells. *Exp. Cell Res.* 339 163–173. 10.1016/j.yexcr.2015.10.032 26524508

[B87] Giorelli M. Livrea P. Trojano M. (2004). Post-receptorial mechanisms underlie functional disregulation of b2-adrenergic receptors in lymphocytes from Multiple Sclerosis patients. *J. Neuroimmunol.* 155 143–149. 10.1016/j.jneuroim.2004.05.013 15342205

[B16] Giovannoni G. (2017). The neurodegenerative prodrome in multiple sclerosis. *Lancet Neurol.* 16 413–414. 10.1016/S1474-4422(17)30127-8 28504102

[B17] Grailer J. J. Haggadone M. D. Sarma J. V. Zetoune F. S. Ward P. A. (2014). Induction of M2 regulatory macrophages through the beta2-adrenergic receptor with protection during endotoxemia and acute lung injury. *J. Innate Immun.* 6 607–618. 10.1159/000358524 24642449 PMC4159611

[B18] Grisanti L. A. Gumpert A. M. Traynham C. J. Gorsky J. E. Repas A. A. Gao E. (2016). Leukocyte-expressed beta2-adrenergic receptors are essential for survival after acute myocardial injury. *Circulation* 134 153–167. 10.1161/CIRCULATIONAHA.116.022304 27364164 PMC5120587

[B19] Harris K. M. Lu T. Lim N. Turka L. A. (2018). Challenges and opportunities for biomarkers of clinical response to AHSCT in autoimmunity. *Front. Immunol.* 9:100. 10.3389/fimmu.2018.00100 29456529 PMC5801415

[B20] Hilbert T. Bongartz J. Weisheit C. Knufermann P. Baumgarten G. Hoeft A. (2013). Beta2-adrenoceptor stimulation suppresses TLR9-dependent IFNA1 secretion in human peripheral blood mononuclear cells. *PLoS One* 8:e65024. 10.1371/journal.pone.0065024 23724117 PMC3665595

[B21] Hu C. Li J. Zhu Y. Bai C. Zhang J. Xia S. (2013). Effects of Al on the splenic immune function and NE in rats. *Food Chem. Toxicol.* 62 194–198. 10.1016/j.fct.2013.08.038 23978415

[B22] Hudson C. A. Mondal T. K. Cao L. Kasten-Jolly J. Huber V. C. Lawrence D. A. (2005). The dietary supplement ephedrine induces beta-adrenergic mediated exacerbation of systemic lupus erythematosus in NZM391 mice. *Lupus* 14 293–307. 10.1191/0961203305lu2078oa 15864916

[B23] Janig W. Green P. G. (2014). Acute inflammation in the joint: its control by the sympathetic nervous system and by neuroendocrine systems. *Auton. Neurosci.* 182 42–54. 10.1016/j.autneu.2014.01.001 24530113

[B24] Jazdzewski K. Bednarczuk T. Stepnowska M. Liyanarachchi S. Suchecka-Rachon K. Limon J. (2007). beta-2-adrenergic receptor gene polymorphism confers susceptibility to Graves disease. *Int. J. Mol. Med.* 19 181–186. 17143563 PMC2526556

[B25] Jiao K. Niu L. N. Li Q. H. Ren G. T. Zhao C. M. Liu Y. D. (2015). beta2-Adrenergic signal transduction plays a detrimental role in subchondral bone loss of temporomandibular joint in osteoarthritis. *Sci. Rep.* 5:12593. 10.1038/srep12593 26219508 PMC4518212

[B26] Kavelaars A. De Jong-De Vos Van Steenwijk T. Kuis W. Heijnen C. J. (1998). The reactivity of the cardiovascular system and immunomodulation by catecholamines in juvenile chronic arthritis. *Ann. N. Y. Acad. Sci.* 840 698–704. 10.1111/j.1749-6632.1998.tb09608.x 9629296

[B27] Kenney M. J. Ganta C. K. (2014). Autonomic nervous system and immune system interactions. *Compr. Physiol.* 4 1177–1200. 10.1002/cphy.c130051 24944034 PMC4374437

[B28] Keranen T. Hommo T. Moilanen E. Korhonen R. (2017). beta2-receptor agonists salbutamol and terbutaline attenuated cytokine production by suppressing ERK pathway through cAMP in macrophages. *Cytokine* 94 1–7. 10.1016/j.cyto.2016.07.016 28162907

[B29] Khoury S. J. Healy B. C. Kivisäkk P. Viglietta V. Egorova S. Guttmann C. R. (2010). A randomized controlled double-masked trial of albuterol add-on therapy in patients with multiple sclerosis. *Arch. Neurol.* 67 1055–1061. 10.1001/archneurol.2010.222 20837847 PMC2954052

[B30] Kneilling M. Hültner L. Pichler B. J. Mailhammer R. Morawietz L. Solomon S. (2007). Targeted mast cell silencing protects against joint destruction and angiogenesis in experimental arthritis in mice. *Arthritis Rheum.* 56 1806–1816. 10.1002/art.22602 17530709

[B31] Kohr D. Singh P. Tschernatsch M. Kaps M. Pouokam E. Diener M. (2011). Autoimmunity against the beta2 adrenergic receptor and muscarinic-2 receptor in complex regional pain syndrome. *Pain* 152 2690–2700. 10.1016/j.pain.2011.06.012 21816540

[B32] Kolmus K. Tavernier J. Gerlo S. (2015). beta2-Adrenergic receptors in immunity and inflammation: stressing NF-kappaB. *Brain Behav. Immun.* 45 297–310. 10.1016/j.bbi.2014.10.007 25459102

[B33] Koopman F. A. Stoof S. P. Straub R. H. Van Maanen M. A. Vervoordeldonk M. J. Tak P. P. (2011). Restoring the balance of the autonomic nervous system as an innovative approach to the treatment of rheumatoid arthritis. *Mol. Med.* 17 937–948. 10.2119/molmed.2011.00065 21607292 PMC3188868

[B34] Lajevic M. D. Suleiman S. Cohen R. L. Chambers D. A. (2011). Activation of p38 mitogen-activated protein kinase by norepinephrine in T-lineage cells. *Immunology* 132 197–208. 10.1111/j.1365-2567.2010.03354.x 21039464 PMC3050443

[B35] Lamkin D. M. Ho H. Y. Ong T. H. Kawanishi C. K. Stoffers V. L. Ahlawat N. (2016). beta-Adrenergic-stimulated macrophages: comprehensive localization in the M1-M2 spectrum. *Brain Behav. Immun.* 57 338–346. 10.1016/j.bbi.2016.07.162 27485040 PMC5011037

[B36] Lantsova V. B. Gerasimov A. S. Sepp E. K. (2013). The role of ADRB2 in myasthenia: genetic and immunological factors. *Bull. Exp. Biol. Med.* 154 351–353. 10.1007/s10517-013-1948-0 23484198

[B37] Lee S. H. Kwon J. Y. Kim S. Y. Jung K. Cho M. L. (2017). Interferon-gamma regulates inflammatory cell death by targeting necroptosis in experimental autoimmune arthritis. *Sci. Rep.* 7:10133. 10.1038/s41598-017-09767-0 28860618 PMC5579272

[B38] Levine J. D. Coderre T. J. Helms C. Basbaum A. I. (1988). Beta 2-adrenergic mechanisms in experimental arthritis. *Proc. Natl. Acad. Sci. U.S.A.* 85 4553–4556. 10.1073/pnas.85.12.4553 2837769 PMC280469

[B39] Liu H. Li W. Gu W. Kong Y. Yang N. Chen L. (2010). Immunoregulatory effects of carvedilol on rat experimental autoimmune myocarditis. *Scand. J. Immunol.* 71 38–44. 10.1111/j.1365-3083.2009.02347.x 20017808

[B40] Lowin T. Straub R. H. (2015). Cannabinoid-based drugs targeting CB1 and TRPV1, the sympathetic nervous system, and arthritis. *Arthritis Res. Ther.* 17:226. 10.1186/s13075-015-0743-x 26343051 PMC4561168

[B41] Loza M. J. Foster S. Peters S. P. Penn R. B. (2006). Beta-agonists modulate T-cell functions via direct actions on type 1 and type 2 cells. *Blood* 107 2052–2060. 10.1182/blood-2005-08-3265 16278302 PMC1895713

[B42] Lubahn C. L. Lorton D. Schaller J. A. Sweeney S. J. Bellinger D. L. (2014). Targeting alpha- and beta-adrenergic receptors differentially shifts Th1, Th2, and inflammatory cytokine profiles in immune organs to attenuate adjuvant arthritis. *Front. Immunol.* 5:346. 10.3389/fimmu.2014.00346 25157248 PMC4127464

[B43] Makhlouf K. Comabella M. Imitola J. Weiner H. L. Khoury S. J. (2001). Oral salbutamol decreases IL-12 in patients with secondary progressive multiple sclerosis. *J. Neuroimmunol.* 117 156–165. 10.1016/S0165-5728(01)00322-8 11431016

[B44] Malysheva O. Pierer M. Wagner U. Wahle M. Wagner U. Baerwald C. G. (2008). Association between beta2 adrenergic receptor polymorphisms and rheumatoid arthritis in conjunction with human leukocyte antigen (HLA)-DRB1 shared epitope. *Ann. Rheum. Dis.* 67 1759–1764. 10.1136/ard.2007.083782 18267980

[B45] Manni M. Granstein R. D. Maestroni G. (2011). beta2-Adrenergic agonists bias TLR-2 and NOD2 activated dendritic cells towards inducing an IL-17 immune response. *Cytokine* 55 380–386. 10.1016/j.cyto.2011.05.013 21683614 PMC3148409

[B46] Morand E. F. (2018). Systemic lupus erythematosus: stress and the onset of SLE. *Nat. Rev. Rheumatol.* 14 127–128. 10.1038/nrrheum.2018.2 29367692

[B47] Munoz-Rodriguez F. J. Font J. Badia J. R. Miret C. Barbera J. A. Cervera R. (1997). Shrinking lungs syndrome in systemic lupus erythematosus: improvement with inhaled beta-agonist therapy. *Lupus* 6 412–414. 10.1177/096120339700600413 9175030

[B48] Nacu A. Andersen J. B. Lisnic V. Owe J. F. Gilhus N. E. (2015). Complicating autoimmune diseases in myasthenia gravis: a review. *Autoimmunity* 48 362–368. 10.3109/08916934.2015.1030614 25915571 PMC4616023

[B49] Namazi M. R. (2004). The beneficial effect of beta2-blockers on humoral autoimmune disorders. *J. Hypertens.* 22 2397–2398. 10.1097/00004872-200412000-00024 15614035

[B50] Nijhuis L. E. Olivier B. J. Dhawan S. Hilbers F. W. Boon L. Wolkers M. C. (2014). Adrenergic beta2 receptor activation stimulates anti-inflammatory properties of dendritic cells in vitro. *PLoS One* 9:e85086. 10.1371/journal.pone.0085086 24465481 PMC3898911

[B51] Nishii M. Inomata T. Niwano H. Takehana H. Takeuchi I. Nakano H. (2006). β_2_-adrenergic agonists suppress rat autoimmune myocarditis: potential role of β_2_-adrenergic stimulants as new therapeutic agents for myocarditis. *Circulation* 114 936–944. 10.1161/CIRCULATIONAHA.105.607903 16908771

[B52] Padro C. J. Sanders V. M. (2014). Neuroendocrine regulation of inflammation. *Semin. Immunol.* 26 357–368. 10.1016/j.smim.2014.01.003 24486056 PMC4116469

[B53] Padro C. J. Shawler T. M. Gormley M. G. Sanders V. M. (2013). Adrenergic regulation of IgE involves modulation of CD23 and ADAM10 expression on exosomes. *J. Immunol.* 191 5383–5397. 10.4049/jimmunol.1301019 24140643 PMC3842235

[B54] Park S. Kim T. Y. Sim S. Oh H. S. Song E. Kim M. (2017). Association of KCNJ2 genetic variants with susceptibility to thyrotoxic periodic paralysis in patients with graves’. *Dis. Exp. Clin. Endocrinol. Diabetes* 125 75–78. 10.1055/s-0042-119527 28008586

[B55] Pawlak C. R. Jacobs R. Mikeska E. Ochsmann S. Lombardi M. S. Kavelaars A. (1999). Patients with systemic lupus erythematosus differ from healthy controls in their immunological response to acute psychological stress. *Brain Behav. Immun.* 13 287–302. 10.1006/brbi.1999.0553 10600217

[B56] Podojil J. R. Sanders V. M. (2005). CD86 and beta2-adrenergic receptor stimulation regulate B-cell activity cooperatively. *Trends Immunol.* 26 180–185. 10.1016/j.it.2005.02.005 15797507

[B57] Pongratz G. Anthofer J. M. Melzer M. Anders S. Grassel S. Straub R. H. (2014). IL-7 receptor alpha expressing B cells act proinflammatory in collagen-induced arthritis and are inhibited by sympathetic neurotransmitters. *Ann. Rheum. Dis.* 73 306–312. 10.1136/annrheumdis-2012-202944 23505234

[B58] Pongratz G. Straub R. H. (2013). Role of peripheral nerve fibres in acute and chronic inflammation in arthritis. *Nat. Rev. Rheumatol.* 9 117–126. 10.1038/nrrheum.2012.181 23147892

[B59] Pongratz G. Straub R. H. (2014). The sympathetic nervous response in inflammation. *Arthritis Res. Ther.* 16:504 10.1186/s13075-014-0504-2 PMC4396833 25789375

[B60] Pont-Kingdon G. Bohnsack J. Sumner K. Whiting A. Clifford B. Guthery S. S. (2009). Lack of association between beta 2-adrenergic receptor polymorphisms and juvenile idiopathic arthritis. *Scand. J. Rheumatol.* 38 91–95. 10.1080/03009740802541488 19177262 PMC2838190

[B61] Qiao G. Chen M. Bucsek M. J. Repasky E. A. Hylander B. L. (2018). Adrenergic signaling: a targetable checkpoint limiting development of the antitumor immune response. *Front. Immunol.* 9:164. 10.3389/fimmu.2018.00164 29479349 PMC5812031

[B62] Ross A. M. Lee C. S. Lutsep H. Clark W. M. (2018). The Influence of beta-adrenergic receptor kinase-1 on stroke-induced immunodeficiency syndrome. *J. Cardiovasc. Nurs.* 33 E3–E10. 10.1097/JCN.0000000000000481 29601376

[B63] Sanders V. M. (2012). The beta2-adrenergic receptor on T and B lymphocytes: do we understand it yet? *Brain Behav. Immun.* 26 195–200. 10.1016/j.bbi.2011.08.001 21855626 PMC3243812

[B64] Scanzano A. Cosentino M. (2015). Adrenergic regulation of innate immunity: a review. *Front. Pharmacol.* 6:171 10.3389/fphar.2015.00171 PMC4534859 26321956

[B65] Sergienko E. Xu J. Liu W. H. Dahl R. Critton D. A. Su Y. (2012). Inhibition of hematopoietic protein tyrosine phosphatase augments and prolongs ERK1/2 and p38 activation. *ACS Chem. Biol.* 7 367–377. 10.1021/cb2004274 22070201 PMC3288537

[B66] Sharma M. Patterson L. Chapman E. Flood P. M. (2017). Salmeterol, a long-acting beta2-adrenergic receptor agonist, inhibits macrophage activation by lipopolysaccharide from *Porphyromonas gingivalis*. *J. Periodontol.* 88 681–692. 10.1902/jop.2017.160464 28398147

[B67] Simkins T. Crawford R. B. Goudreau J. L. Lookingland K. J. Kaplan B. L. (2014). Enhanced humoral immunity in mice lacking CB1 and CB2 receptors (Cnr1^-/-^/Cnr2^-/-^ mice) is not due to increased splenic noradrenergic neuronal activity. *J. Neuroimmune Pharmacol.* 9 544–557. 10.1007/s11481-014-9549-x 24870806

[B68] Slota C. Shi A. Chen G. Bevans M. Weng N. P. (2015). Norepinephrine preferentially modulates memory CD8 T cell function inducing inflammatory cytokine production and reducing proliferation in response to activation. *Brain Behav. Immun.* 46 168–179. 10.1016/j.bbi.2015.01.015 25653192 PMC4414741

[B69] Soliven B. Rezania K. Gundogdu B. Harding-Clay B. Oger J. Arnason B. G. (2009). Terbutaline in myasthenia gravis: a pilot study. *J. Neurol. Sci.* 277 150–154. 10.1016/j.jns.2008.09.033 18952242

[B70] Sugaya K. Nishijima S. Yamada T. Miyazato M. Hatano T. Ogawa Y. (2002). Molecular analysis of adrenergic receptor genes and interleukin-4/interleukin-4 receptor genes in patients with interstitial cystitis. *J. Urol.* 168 2668–2671. 10.1016/S0022-5347(05)64241-3 12442007

[B71] Takenaka M. C. Araujo L. P. Maricato J. T. Nascimento V. M. Guereschi M. G. Rezende R. M. (2016). Norepinephrine controls effector T cell differentiation through beta2-adrenergic receptor-mediated inhibition of Nf-kappab and AP-1 in dendritic cells. *J. Immunol.* 196 637–644. 10.4049/jimmunol.1501206 26663782

[B72] Tandale A. Joshi M. Sengupta D. (2016). Structural insights and functional implications of inter-individual variability in beta2-adrenergic receptor. *Sci. Rep.* 6:24379. 10.1038/srep24379 27075228 PMC4830965

[B73] Tracey K. J. (2014). Lymphocyte called home: beta2-adreneric neurotransmission confines T cells to lymph nodes to suppress inflammation. *J. Exp. Med.* 211 2483–2484. 10.1084/jem.21113insight3 25512581 PMC4267244

[B74] Tsai C. P. Lin F. C. Lee C. T. (2014). Beta2-adrenergic agonist use and the risk of multiple sclerosis: a total population-based case-control study. *Mult. Scler.* 20 1593–1601. 10.1177/1352458514528758 24732071

[B75] Ulloa L. Quiroz-Gonzalez S. Torres-Rosas R. (2017). Nerve stimulation: immunomodulation and control of inflammation. *Trends Mol. Med.* 23 1103–1120. 10.1016/j.molmed.2017.10.006 29162418 PMC5724790

[B76] Wahle M. Kolker S. Krause A. Burmester G. R. Baerwald C. G. (2001). Impaired catecholaminergic signalling of B lymphocytes in patients with chronic rheumatic diseases. *Ann. Rheum. Dis.* 60 505–510. 10.1136/ard.60.5.505 11302874 PMC1753636

[B77] Wahle M. Krause A. Pierer M. Hantzschel H. Baerwald C. G. (2002). Immunopathogenesis of rheumatic diseases in the context of neuroendocrine interactions. *Ann. N. Y. Acad. Sci.* 966 355–364. 10.1111/j.1749-6632.2002.tb04235.x 12114292

[B78] Wang L. Zhang Y. He M. (2017). beta2-Adrenergic receptor gene polymorphisms in the relapse of myasthenia gravis with thymus abnormality. *Int. J. Neurosci.* 127 291–298. 10.1080/00207454.2016.1202952 27338803

[B79] Wei W. (2016). Soft regulation of inflammatory immune response. *Chin. Pharmacol. Bull.* 32 297–303.

[B80] Weissert R. (2017). Adaptive immunity is the key to the understanding of autoimmune and paraneoplastic inflammatory central nervous system disorders. *Front. Immunol.* 8:336. 10.3389/fimmu.2017.00336 28386263 PMC5362596

[B81] Woo A. Y. Jozwiak K. Toll L. Tanga M. J. Kozocas J. A. Jimenez L. (2014). Tyrosine 308 is necessary for ligand-directed Gs protein-biased signaling of beta2-adrenoceptor. *J. Biol. Chem.* 289 19351–19363. 10.1074/jbc.M114.558882 24831005 PMC4094047

[B82] Wu H. Chen J. Song S. Yuan P. Liu L. Zhang Y. (2016). beta2-adrenoceptor signaling reduction in dendritic cells is involved in the inflammatory response in adjuvant-induced arthritic rats. *Sci. Rep.* 6:24548. 10.1038/srep24548 27079168 PMC4832233

[B83] Xu B. Y. Arlehag L. Rantapaa-Dahlquist S. B. Lefvert A. K. (2005). beta2 Adrenoceptor gene single nucleotide polymorphisms are associated with rheumatoid arthritis in northern Sweden. *Ann. Rheum. Dis.* 64 773–776. 10.1136/ard.2004.027532 15498794 PMC1755488

[B84] Zalli A. Bosch J. A. Goodyear O. Riddell N. Mcgettrick H. M. Moss P. (2015). Targeting ss2 adrenergic receptors regulate human T cell function directly and indirectly. *Brain Behav. Immun.* 45 211–218. 10.1016/j.bbi.2014.12.001 25526818

[B85] Zhang Z. W. Qin X. Y. Che F. Y. Xie G. Shen L. Bai Y. Y. (2015). Effects of beta 2 adrenergic agonists on axonal injury and mitochondrial metabolism in experimental autoimmune encephalomyelitis rats. *Genet. Mol. Res.* 14 13572–13581. 10.4238/2015.October.28.17 26535670

[B86] Zhao W. Tong T. Wang L. Li P. P. Chang Y. Zhang L. L. (2011). Chicken type II collagen induced immune tolerance of mesenteric lymph node lymphocytes by enhancing beta2-adrenergic receptor desensitization in rats with collagen-induced arthritis. *Int. Immunopharmacol.* 11 12–18. 10.1016/j.intimp.2010.09.018 20955833

